# MicroRNA Mediated Plant Responses to Nutrient Stress

**DOI:** 10.3390/ijms23052562

**Published:** 2022-02-25

**Authors:** Waqar Islam, Arfa Tauqeer, Abdul Waheed, Fanjiang Zeng

**Affiliations:** 1 Xinjiang Key Laboratory of Desert Plant Roots Ecology and Vegetation Restoration, Xinjiang Institute of Ecology and Geography, Chinese Academy of Sciences, Urumqi 830011, China; drwaheed@ms.xjb.ac.cn; 2 State Key Laboratory of Desert and Oasis Ecology, Xinjiang Institute of Ecology and Geography, Chinese Academy of Sciences, Urumqi 830011, China; 3 Cele National Station of Observation and Research for Desert-Grassland Ecosystems, Cele 848300, China; 4 University of Chinese Academy of Sciences, Beijing 100049, China; 5 College of Horticulture, FAFU-UCR Joint Center and Fujian Provincial Key Laboratory of Haixia Applied Plant System Biology, Fujian Agriculture and Forestry University, Fuzhou 350002, China; arfa.tauqeer@yahoo.com

**Keywords:** abiotic stresses, environmental constraints, genetic regulations, major nutrients, plant nutrition, arid environment

## Abstract

To complete their life cycles, plants require several minerals that are found in soil. Plant growth and development can be affected by nutrient shortages or high nutrient availability. Several adaptations and evolutionary changes have enabled plants to cope with inappropriate growth conditions and low or high nutrient levels. MicroRNAs (miRNAs) have been recognized for transcript cleavage and translational reduction, and can be used for post-transcriptional regulation. Aside from regulating plant growth and development, miRNAs play a crucial role in regulating plant’s adaptations to adverse environmental conditions. Additionally, miRNAs are involved in plants’ sensory functions, nutrient uptake, long-distance root transport, and physiological functions related to nutrients. It may be possible to develop crops that can be cultivated in soils that are either deficient in nutrients or have extreme nutrient supplies by understanding how plant miRNAs are associated with nutrient stress. In this review, an overview is presented regarding recent advances in the understanding of plants’ responses to nitrogen, phosphorus, potassium, sulfur, copper, iron, boron, magnesium, manganese, zinc, and calcium deficiencies via miRNA regulation. We conclude with future research directions emphasizing the modification of crops for improving future food security.

## 1. Introduction

The normal development and growth of plants requires at least 17 elements. The mineral nutrients comprise 14 of the 17 essential nutrients, the others being oxygen, carbon, and hydrogen [1]. To obtain optimum tissue and cellular development, the concentrations level of these mineral nutrients should be managed with in limited physiological ranges after absorption by plant roots. However, the nutrient compositions of agricultural systems or natural habitats vary, and some must make up for severe nutrient deficiencies [2]. Micronutrients and macronutrients that are not available in enough or excessive amounts can cause nutrient stress. This is rapidly becoming the most serious environmental stress, which has a negative impact on normal growth and development [3]. According to researchers, plants have evolved efficient mechanisms to observe their nutritional status and adapt to variations in nutrient concentrations [4,5,6]. With the success of the genome sequencing of plants and through the development of new genomic tools, several regulatory elements have been identified as being involved in plant responses to nutritional stress [7,8]. These elements, which include nutrient transporter protein, transcriptional factors, rib regulators, and associated proteins, create a complex regulation system that allows the plant to detect nutrient stress and modify itself in response by altering a wide range of physiochemical, biological, structural, and biochemical mechanisms [9].

**Figure 1:**
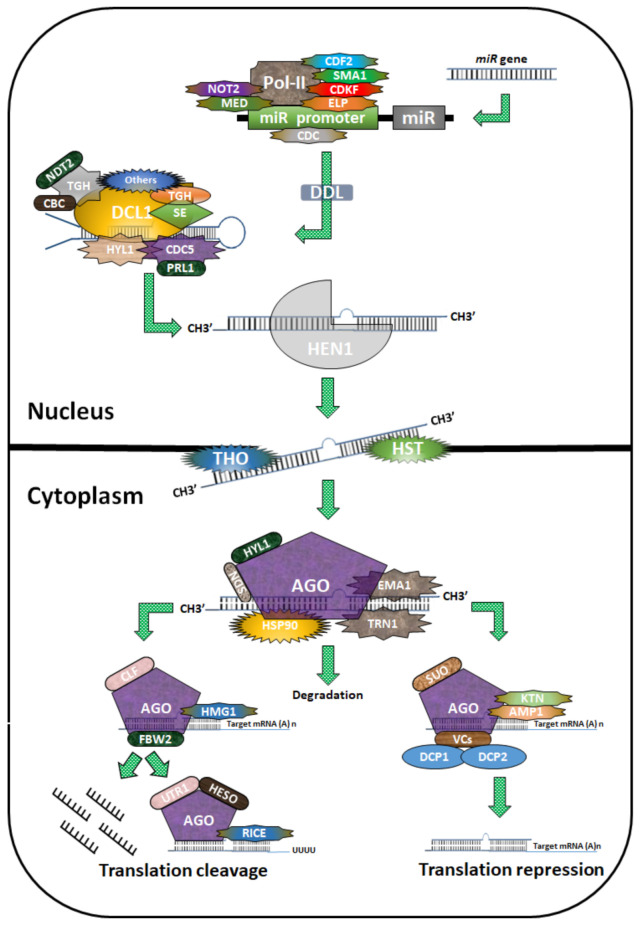
Biogenesis of miRNAs in plants. The diagram depicts miRNA biogenesis in plants. Pol II first transcribes miR genes into pri-miRNAs, leading to the formation of a hairpin structure. The process is regulated by cycling DOF transcription factors (CDF2), SMALL1 (SMA1), cyclin-dependent protein kinase (CDKF), extension gene (ELP), a protein containing the MYB domain (CDC), Mediator2 (MED2), and NOT2. During nuclear splicing and processing, the cap-binding protein complex (CBC including CBP20 and CBP80HYL1), PRL1 (an evolutionarily conserved WD-40 protein), DDL, TGH, and SERRATE (SE) paly regulatory roles. Dicer-Like 1 (DCL1) progressively processes pri-miRNAs and pre-miRNAs to produce one or more phased miRNA/miRNA* duplexes, which are methylated by HUA enhancer 1 (HEN1) and delivered to the cytoplasm by HST1 (HASTY). The miRNA is chosen and integrated into a specific argonaute1 (AGO1)-containing RISC (RNA-induced silencing complex), which guides translation inhibition or cleavage of the target mRNA transcript.

In recent years, miRNAs have been found to be involved to plant stress responses through the post-transcriptional regulation of a variety of transcription factors (TFs) [10,11,12,13]. miRNAs are small, intracellular solitary, non-coding RNAs (19 to 24 base pairs), and they are recognized to mediate endogenous expression. They are derived from single stranded (SS)-RNA and have a helical secondary structure [14,15]. Plants usually transcribe miRNAs from separate *MIR* genes by RNA polymerase II interactions, resulting in 5’ capped, 3’ polytailed miRNA molecules (Figure 1) [16]. Dicer-like 1 (DCL1) proteins synthesize miRNAs from pri-miRNAs to create catalyst miRNAs, which are then recognized by other DCL1 proteins, which go on to form miRNA/miRNA* duplexes [17]. The RNA-induced silencing complex (RISC) catalyzes the associations of miRNAs with target transcripts using an argonaute (AGO) protein, which is incorporated into a duplex miRNA strand. This helps the AGO to attach to targets through sequence complementarity. The miRNA* strand is often weakened after the fully-developed strand is released [16].

Plant growth and development are regulated by miRNAs [14]. Plants use miRNAs to respond to a variety of biotic and abiotic stressors, such as nutritional stress [18]. Plant miRNAs have been shown to be sensitive to a wide range of nutritional stressors, including nitrogen, phosphorus, potassium, sulfur, copper, iron, boron, magnesium, manganese, zinc, and calcium deficiencies and fluctuations (Table 1). miRNAs that are responsive to nutrient stress and their corresponding targets are summarized in this review. Moreover, miRNAs are described briefly in the context of their regulatory role in modulating the responses of plants to nutrient stress.

## 2. microRNAs in Nutrient Stress

### 2.1. miRNAs and Nitrogen Stress

Nitrogen (N) is an important factor mainly found in amino acid residues, peptides, nucleotides, cofactors, and a wide variety of secondary plant substances; therefore, it is vital for plant development and rehabilitation [19]. Even though N is present in soil in a variety of forms, plant roots absorb N as nitrates and ammonium in a systematic manner [20]. The accessibility of N to plant root systems is commonly a critical barrier for growing plants and the yields of crops [21]. Plants have developed a number of strategies to adapt to variations in N availability in soil, including structural, physical, and metabolic adaptations [22]. A clear link between inorganic N transporters and cell wall construction has been established via a variety of co-expressed remodeling enzymes. Pectin and xyloglucan production enzymes were shown to be significantly co-regulated with N transporters, implying a link between N assimilation and cell wall growth regulation [23].

miRNA functions have been investigated in response to nitrate and N deficiency in the past few years. In response to N, miR167 and miR393 restrict root growth [24]. Moreover, several other N starvation responsive miRNAs have been discovered in plants using in situ hybridization, small RNA (sRNAs) sequencing at high efficiency, real-time quantitative reverse transcription-polymerase chain reaction (qRT-PCR), and hybridization microarray analyses (Table 1). In cotton, miR167 is a well-known regulated miRNA that targets two auxin response factors (ARFs), i.e., ARF6 and ARF8 [25]. Based on microarray hybridization of roots, it was shown that ARF8 was expressed in pericyclic and horizontal root cap cells of nitrate-treated *Arabidopsis* roots [26]. Utilizing qRT-PCR and β-glucuronidase (GUS) fusion analysis, it was concluded that nitrogen induces ARF8 and inhibits miR167. Additionally, it was discovered that ARF8-GUS binding to a mutant miR167-binding site has ongoing negative effects on nitrate regulation. This evidence supports the theory that miR167 suppression causes ARF8 accumulation in pericycle cellular process in response to nitrate analysis [27]. Nitrate regulation of lateral root emergence was completely lost in genetically modified plants expressing miR167 and ARF8 blank mutants. Consequently, the miR167 or ARF8 module controls adventitious root plants’ responses to N, and even more so, N metabolic enzymes that are produced by downstream of nitrification and absorption [24]. Furthermore, it was discovered that miR167 is extra sensitive to N deficiency in maize [28] and in *Arabidopsis* [29] indicating that miR167 has vital roles in monocots and dicots’ adaptation to N-limited conditions.

miR393 was discovered to be stimulated by nitrate via eliminating the N oxidase null mutations. Furthermore, activation of miR393 was observed by glycine and ammonium nitrate [30]. Research findings indicate that miR393 is activated by the N signal transmitted during nitrification and absorption [31]. miR393 specifically targets the basic helix–loop–helix (bHLH) signaling pathway bHLH77 and the phytohormones sense organs, e.g., auxin signaling F-box proteins (AFB3, AFB2) and TIR1 (Toll/interleukin-1 receptor) [32]. Individually, the phytohormone sensing organ AFB3 is influenced through miR393-mediated N treatment. Moreover, nitrate regulates auxin-receptive and auxin associated genes that are uninfluenced by auxin treatment, including ARF18 and ARF9 [33]. Furthermore, nitrate had no effect on main root development in miR393-overexpressing plants or afb3-1 mutants, meaning that the miR393/AFB3 regulation module is essential for modifying root development and for the response to N deficiency [34]. In addition to main root development, the miR393/AFB3 element controls horizontal root development in the reaction to nitrate treatment. These findings show that miR393 and AFB3 control nitrate-produced variations in root structure, potentially through the auxin-signaling pathway.

It was discovered that N deficiency significantly downregulates Arabidopsis miR169, and target nuclear transcription factor Y subunit-alpha (*NFYA*) genes, *NFYA8*, *NFYA5*, and *NFYA2* [29,35]. Other research found that miR169a is downregulated by N deficiency in Arabidopsis shoots and roots, and that it is essential under N-limited conditions, for the regulation of *NFYA* expression [36,37] (Figure 1). When compared to wild-type plants, transgenic plants that overexpressed miR169a acquired less N and were more susceptible to N deficiency. It has been shown that extreme susceptibility to N uptake occurs in miR169a-overexpressing plants, and that this is strongly correlated with inhibition of nitrate transport system (*NRT*) genes, *AtNRT2.1* and *AtNRT1.1*, by the TF called NF-YA [29]. The miR169 gene has also been found in soybeans, maize, and wheat, which contributes to plant tolerance to fluctuations in N levels [38].

Using various methods, many N-responsive miRNAs in plant species have been discovered (Table 1). qRT-PCR analysis in Arabidopsis indicated that some pri-miR169s and pri-miR398a are suppressed by N deficiency [39]. Next-generation sequencing data revealed that nine different types of miRNA were suppressed by nutrient deficiency in Arabidopsis; and five miRNA families were stimulated, including miR857, miR398, miR397, and miR395 [40]. Nine novel miRNAs that are responsive to nutrient deficiency were discovered [29]. According to molecular marker techniques, 15 miRNA types were discovered to be overexpressed in rice, and susceptibility to low N is determined by miRNAs, showing miRNAs’ importance [41]. Several research groups have discovered N-receptive miRNAs in maize [42,43,44,45] (Table 1). There are 14 miRNA types in maize that are susceptible to transiently or chronically low N levels, as determined using two genotyping systems [45]. Five preserved families (miR171, miR528, miR395, miR827, and miR169) differentially expressed in maize were identified using northern blot analysis [44]. The N-deficient maize plants were found to downregulate six miRNAs, including miR408, miR169, miR166, miR528, miR528*, and miR169*, using genotype, qRT-PCR, and in situ hybridization analysis in maize [42]. Most of the genes assumed to be targeted by N-responsive miRNAs are translated by plants growth. They are involved signal transmission, nutrient composition, and oxidation pressure susceptibility, indicating that these miRNAs can be associated with managing numerous physical reactions in response to N deficiency (Table 1).

### 2.2. miRNAs and Phosphate Stress

Phosphate (P) is the most important inorganic nutrient for root development and efficiency. Other than being a structural element of basic organic compounds such as nucleotides and phosphatide, P is also linked to energy transmission, energy production, metabolism, and amino acid synthesis in plants. [46]. There is a limited amount of P in soil that is available to plants by absorption through the soil and rainfall/transformation into biological forms [46]. Plants often alter their root morphologies and architectures to manage low P stress followed by root cell wall adaptations. Cell wall proteins have been shown to play important roles in the synthesis of cell walls, transmission of signals, and protecting of cells from environmental stress [47].

**Figure 2:**
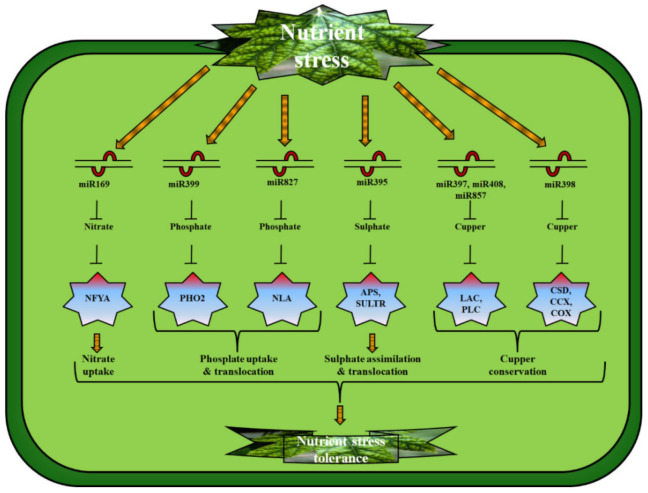
Plant microRNAs: their genetic targets and corresponding functions that induce tolerance against nutrient stress. miR169. miR399, miR827, miR395, miR397, miR408, MiR857, and miR398 respond to N, P, S, and Cu stress via targeting *nuclear transcription factor Y subunit-alpha (NFYA), phosphate starvation-responsive gene (PHO2), nitrogen limitation adaptation (NLA), ATP sulfurylase (APS), sulfate transporter (SULTR), laccases (LAC), cold shock domain (CSD)*, and *cyclooxygenase (COX)* to regulate N uptake, P uptake and translocation, S assimilation, and Cu conservation in plants, respectively, thereby having active roles in nutrient stress tolerance.

Recently, it was discovered that miRNAs perform regulatory roles in plants’ responses to P deficiency. By downregulating its target gene, ubiquitin C *(UBC24)* (also known as phosphate starvation-responsive gene, *PHO2*), miR399 controls P metabolism by P addition, division, and demobilization [48]. *UBC24* codes an E2 regulatory protein-associated enzyme with miR399 target sites in its 5’ UTR [49]. miR399 was discovered to be firmly and precisely upregulated by P deficiency, suggesting that P deficiency inhibits the expression of *UBC24* [50]. Upregulation of miR399 in Arabidopsis enhances P intake and distribution to the shoot, resulting in an overabundance of P in the shoot [51]. Genetically modified rice (specifically, *Oryza sativa* spp.) transcriptionally upregulating osa-miR399f or osa-miR399j showed similar phenotypes [52]. Additionally, it was found that heterologous upregulation of Arabidopsis miR399 in tomato resulted in improved P availability in both roots and shoots, improved transport of protons from roots, and improved proton excretion from shoots [53]. Characteristics of miR399-upregulating plants and ubc24-T-DNA mutants equally represent the characteristics of a pho2 mutant [54]. Phosphate slows and suppresses P starvation-induced *PSI* genes in pho2 mutants or miR399-upregulating plants, which consist of *IPS1 (insensitive to phospate starvation 1)* and the P carrier genes, further showing that PHO2 deficiency resembles P starvation, both of which contribute significantly to P over-abundance in shoots at below adequate P situations [55,56] (Figure 2). Although the PHO2-moderated decomposition of PHO1 on the endomembrane is necessary to regulate P homeostasis, other unidentified aspects controlled by PHO2 may occur to account for the toxic effects of P on physical composition [57]. Phosphate starvation response-1 (PHR1), PHR1-like 1 (PHL1), and MYB (myeloblastosis) transcription regulators are regulators of P deficiency, as miR399 restricts miR399 transcription and its activity [58]. Phr1-mutants could not upregulate miR399 during P deficiency, showing that PHR1 may suppress miR399 by attaching to P1BS in promoter regions [59]. Recently, it was discovered that Arabidopsis miR399f is controlled by the P deficiency protein *IPS1* and is not carved from miR399, resulting in less movement of miR399, thereby protecting PHO2 transcription from division [60].

In Arabidopsis, miR827 plays a specific role in P homeostasis by inhibiting the expression of *NLA (nitrogen limitation adaptation)* (Figure 2). A 2-NLA mutant exhibited suppression of P transport-related genes, i.e., *PHD finger protein 1 (PHF1)* and *phosphate transporter 1 (PHT1)*. An NLA mutant’s initial maturating phenotype was considered to be affected by extreme P deposition in limited N conditions. NLA functions as a nutrient-dependent mechanism for maintaining P homeostasis [61]. In addition, NLA mutant plants presented morphologies like those of P-poisoned plants. Remarkably, the P concentrations in the miR827 variant and the NLA-upregulating plants were comparable [61]. Arabidopsis miR827 responded similarly to rice miR827 when P deficiency was present. The *major facilitator superfamily (OsSPX-MFS1* and *OsSPX-MFS2)* proteins are formed by dividing two *SPX-MFS*. Astonishingly, P detection is accomplished by two different OsSPX-MFS-encoding genes; *OsSPX-MFS1*, and *OpSPX-MFS2*, which have indicated opposite reactions to P deficiency [62]. Moreover, it was observed that osspx-mfs1 mutants improved P concentrations by reducing P remobilization from old to matured leaves, indicating that the miR827/*SPX-MFS1* element controls the P balance in rice plants [62]. Numerous other plant species, including Arabidopsis [63], maize [64], white lupine [65], soybean [66,67], and wheat [68], have been shown to contain P-responsive microRNAs (Table 1).

### 2.3. miRNAs and Potassium Stress

In addition to photosynthesis, osmoregulation, and enzyme activation, potassium (K) is critical to many plant processes, such as cell turgor regulation, cellular expansion, modulation of the cell membrane’s electric potential, and balancing of the pH. It influences transcription and post-transcriptional processes. It can be used to maximize crop yields, as it is an important component for plant growth and development [69,70]. Though it is possible that certain cellular mechanisms which are involved have been uncovered, the morphological and physiological adaptations used by plants to cope with K deficiency remain a mystery. In order to maximize crop yield and quality, further research into K deficiency’s effects on plants is necessary, including identifying the mechanisms leading to the observed changes.

Although some studies suggest certain miRNAs are involved in signal transduction in plants, the exact mechanisms by which they regulate K uptake is unknown. Specifically, the *dormancy-associated MADS-box (OsMADS23)* target gene was significantly upregulated in K deficiency, and Osa-miR444a apparently regulates both N and P accumulations [71]. A further consequence of the low K conditions was the induction of Hvu-miR319, which repressed *growth response factor (HvGRF)* expression, which promoted Hvu-miR396 transcription in barley [72]. A number of miRNAs have shown differential expression under low-K stress, including Hvu-miR160a, Hvu-miR169h, and Hvu-miR396c, which are implicated in regulating different photosynthetic processes [73].

Recently, researchers have disclosed that cotton and wheat’s miRNA expression was altered by low dietary K availability (Table 1). On the other hand, the K deficiency resulted in altered expression of 16 out of 20 miRNAs, four days and at eight days after transfection, the exceptions being miR393, miR395, miR396, and miR778 [74,75]. In response to K deficiency, wheat may increase root growth and nutrient uptake through molecular mechanisms. In peanut plants, root development is influenced by miRNAs that play critical roles in K deficient conditions. miR156 and miR390 have been proposed to be upregulated in K deficiency, along with miR160, miR164, and miR393. A miRNA-mediated pathway and mechanism may be responsible for peanuts’ responses to N and K deficiency stresses [76].

RISC modulates the regulatory pathway by AGO1 in tomatoes via miR168. There has been no conclusive evidence pointing to the function of miR168 in regulating AGO1 in tomatoes under K deficiency stress. SlmiR168 and its target gene expression differ among tomato plants tolerant of low K (JZ34) and those sensitive to low K (JZ18). Pri-SlmiR168-expressing transgenic tomato plants demonstrated superior plant development and K content in roots in a K-deficit environment to non-transgenic or wild-type tomato plants. It was observed that 35S:rSlAGO1 tomatoes exhibited differential upregulation of various miRNAs as compared to wild type tomatoes. In 35S:SlmiR168a plants, miRNA levels were much lower than in WT plants. The root growth and cytokinin (CTK)/abscisic acid (ABA) pathways were regulated by 12 miRNAs/mRNAs that were identified in the integrated analysis. Low-K stress enhances the development of plants via SlmiR168 regulation of SlAGO1A. This regulation mechanism affects CTK/ABA and root growth modulation pathways [77].

The targets of miRNAs detected in Tibetan wild barley cultivated two days or seven days after low-K stress have been identified through bioinformatic predictions and degradome analysis. In total, 65 miRNAs were identified that expressed differently under low-K stress. miR164c, miR169h, and miR395a modules are able to communicate with the tricarboxylic acid (TCA) cycle and other important pathways, i.e., glycolysis and pentose phosphate pathways. Low-K stress appears to regulate Ca^2+^ signaling through osa-miR166 and ghr-miR482. These miRNAs are thought to be involved in the ethylene production process in plants growing in low-K environments. Some miRNAs implicated in photosynthetic regulation under low-K stress, such as miR160a, miR396c, and miR169h, differed across two barley genotypes, implying that these selectively expressed miRNAs and their targets are critical for plants in low-K environments. [73].

### 2.4. miRNAs and Sulfate Stress

Many plant metabolites require sulfate (S), including carbohydrates and proteins, sulfolipids, and micronutrients, which are all important for physiological functions [78]. Following the significant reductions in anthropogenic sulfur emissions, less S accessibility in top soil limits plant growth [79]. The most common sulfur form consumed and translocated into different tissues for absorption is inorganic S. Sulfate uptake from topsoil is primarily accomplished through two high-affinity S carriers, SULTR1;1 (sulfate transporter1;1) and SULTR1;2 (Sulfate transporter1;2). Low-affinity S carriers, such as SULTR2;1, SULTR2;2, and SULTR3;5, are used in plants during S transfer [80]. Sulfate acclimatization occurs via plasmidic ATP sulfurylase (APS), which can be deposited in vesicles or absorbed in chloroplasts [81].

A sulfate deficit affects miR395 while targeting the *APS1* and *APS3* genes (and the *SULTR2-1* gene) directly [82] (Figure 2). All six Arabidopsis miR395 genes have been identified that are induced by S starvation using transgenic genetically modified (GM) plants containing miR395 promoter–GFP reporter [83]. The overexpression of miR395 resulted in the downregulation of the *APS4*, *APS1*, and *SULTR2-1* transcripts after S deprivation [82,83]. Tissue-specific occurrence results show that miR395 was identified largely in phloem associated cells, whereas SULTR2-1 is found predominantly in cells of the xylem [83,84]. miR395 thus has the ability to limit SULTR2-1 expression in the xylem in the absence of SULTR2-1. miR395 can overcome articulation of SULTR2-1 in phloem companion cells, which may subsequently promote S uptake in the phloem from roots to shoots while inhibiting shoot-to-root transfer in the xylem (Figure 2) [84]. The TF named *SLIM1* (Sulphur limitation 1) belongs to the EIL (ethylene-insensitive-like) family. It regulates miR395 expression [85]. Sulfate deficit stimulates *SLIM1*; one of the critical regulators of the S starvation reaction, *SULTR1-2*; and other S-starvation response genes. In contrast to miR395 activation in wild-type plants, slim1 mutants treated by S deprivation showed no significant differences in miR395 expression [85]. Activation of miR395 by SLIM1 is essential for enhanced S transfer from roots to shoots, ensuring effective S incorporation in the shoots. Though it is unknown whether *SLIM1* regulates miR395 accumulation directly or indirectly, recent evidence for miR395’s regulatory role in S hybridization and assimilation has been reported [84]. Despite showing S deficiency symptoms, miR395-overexpressing plants showed higher S concentrations in their shoots than land race plants. These S over-accumulator mutant plants’ paradoxical phenotype of S deficiency could be caused by S assimilation and S relocation between leaves. The fact that miR395-overexpressing plants have characteristics like the aps1-1-sultr2 triple mutant endorses this theory. Findings show that miR395 influences S transport between leaves by cleaving *SULTR2* and regulates S deposition in the shoot via targeting *APS* genes (Figure 2) [83]. Furthermore, miR395 was shown to respond to metabolites that control S absorption, indicating that miR395 may possibly have a role in the S assimilation regulatory network. The molecular process that controls the expression of miR395 in the S integration pathway, however, remains unknown [86]. There is evidence that MiR395 has been detected in the genomes of *Sorghum bicolor, Brassica napus, O. sativa, Medicago truncatula, and Solanum lycopersicum* [38,87,88]. The regulatory pathway miR395/*APS-SULTR2* is now known to be maintained in plants. Lack of S alters expression of miR156, miR160, miR164, miR167, miR168, and miR394 in *B. napus* [89], in addition to miR395, implying that they are involved in S adaptation (Table 1).

### 2.5. miRNAs and Copper Stress

Many metal proteins require copper (Cu) as a cofactor, including plastocyanin, superoxide dismutase (SOD), laccases (LAC), and cytochrome C oxidase [90]. A Cu shortage in plants causes a variety of issues, including restricted growth and production. On the other hand, Cu accumulation limits plant development and affects cellular activities, such as photosynthetic electron transport [91]. Several molecular pathways involved in cell wall development and other cellular dynamics include chloroplastic and mitochondrial Cu transport and homeostasis [92]. Numerous approaches for regulating Cu homeostasis in plants have evolved, involving a diverse set of proteins and genes [91]. Plants have been found to respond to Cu deficiencies through miRNAs (miR398, miR397, miR408, and miR857) [93]. miR398, is articulated by three Arabidopsis miR398 genes; *cyclooxygenase (COX5b-1), cold shock domain (CSD1* and *CSD2),* encoding subunits of mitochondrial cytochrome C oxidase; and *Cu chaperone for SOD* (*CCS1* and *CSD2*, encoding cytosolic and chloroplast CSD, respectively) [90,93,94]. Cu deficiency significantly increases miR398 expression. *CSD1* and *CSD2* are its target genes, which are reduced [94] (Figure 2). Cu deficiency also activates Fe-SOD, which substitutes *CSD* functionally. Reduced Cu availability for other Cu proteins such as plastocyanin may result from reducing Cu-containing protein CSD [95]. Nevertheless, when exposed to high Cu stress, miR398 expression decreases, because of which *CSD1* and *CSD2* are induced post-transcriptionally [94] (Figure 2). When exposed to significant Cu stress, through *CSD*, superoxide radicals can be effectively purified and Cu protein synthesis can be increased. GM crops expressing an amiR398-resistant version of *CSD2* are hence significantly extra resistant to oxidative stresses, such as great Cu strain [94].

miR398 plays an important role in the regulation of Cu homeostasis by downregulating the non-significant Cu proteins when Cu concentrations are low or high (Table 1). Non-essential Cu proteins, including laccases and plastocyanin, have also been discovered to be targeted by miR397, miR408, and miR857 [95,96]. These three miRNAs, like miR398, were enhanced by Cu stress, and their appearance patterns were found to be inversely linked with those of their corresponding target genes [95]. Moreover, these three supplementary Cu-responsive miRNAs can aid in the optimization of Cu supply for the critical Cu-comprising TF in Arabidopsis, i.e., SPL7 (SQUAMOSA promoter binding protein-like 7) [97]. Similarly, SPL7 has been demonstrated to regulate miR397, miR398, miR408, and miR857 expression as well [97]. A number of studies have shown that SPL7 binds to its target, the miR398 promoter, which is necessary and sufficient for the in vivo response to Cu deficiency. Unlike miR398b and miR398c, miR398a’s promoter sequence lacks the GTAC motifs, which could explain why miR398a has a lower expression level and is less sensitive to Cu shortage. However, miR398 and miR398c, the spl7 mutant, had considerably decreased echelons of miR397a, miR408, and miR857, demonstrating that SPL7 stimulates Cu-responsive miRNA transcription. SPL7 appears to be a key player in regulating Cu proteins, including laccases, plastocyanin, and Cu/Zn SOD, through Cu-receptive miRNAs [97].

### 2.6. miRNAs and Boron Stress

Boron (B) is required for appropriate plant growth and development. It participates in a variety of physiological activities, including cell wall maintenance, lipid metabolism, cell division, protein and nucleic acid metabolism, and cell division [98]. A few studies have found that miRNAs are involved in B-stress responses (Table 1). For example, the B-stress responsive miRNA network and its corresponding pathways have been studied in barley [99]. Furthermore, the researchers compared the miRNA profiles of root and leaf samples. The existence of 31 recognized and three novel miRNAs in barley was studied, with 25 of them demonstrating a B-treatment response. In certain tissues, miRNAs expressed specifically; e.g., miR156, miR171, miR397, and miR444 were only expressed in leaves. The miRNAs specifically targeted and degraded 934 barley transcripts. When computing the target genes of miRNAs, in silico analysis uncovered that many of the miRNA target genes were conserved, including TFs such as SPLs, ARFs, and MYBs. Most of these target genes were related to plant development and responses to environment changes. Several miRNAs in barley, including miR408, may play critical roles in protecting the plant from B exposure [99]. Furthermore, qRT-PCR analysis of French beans (*Phaseolus vulgaris* L.) exposed to high levels of B revealed differences in miRNA expression. miRNA targets were also studied for their impacts on gene expression during B stress. It was confirmed by GO (Gene Ontology) that plant miRNAs have roles in a number of cellular activities, including the circadian cycle and vegetative development [100].

B deficiency is a problem that is widely prevalent among citrus trees. Citrus species have been shown to contain B-responsive miRNAs in a few studies. The results of high-throughput Illumina sequencing (HTIS) in *Citrus sinensis* roots revealed 52 upregulated and 82 downregulated miRNAs. This demonstrates roots’ amazing metabolic plasticity, which may help plants tolerate B deprivation [101]. It was proposed that several aspects of miRNAs may affect the adaptation of roots to B-deficiency such as (a) upregulating miR474 and downregulating miR782 and miR843 to scavenge reactive oxygen species (ROS); (bboosting the expression of miR394 and lowering the expression of miR5023, thus making cells more sensitive to B-deficiency; (c) downregulating miR830, miR5266, and miR3465 transcripts to improve fluid transport in cells; (d) regulating osmo-protection via miR474 and other metabolic reactions through miR5023 and miR821. It was exhibited that the expression of other miRNAs, including miR472 and miR2118 in roots, was decreased as B-deficiency occurred, resulting in the reduction of diseases resistance genes leading towards the decrease in root disease resistance [101]. Similarly, RNA transcript analysis in trifoliate orange (*Poncirus trifoliata*) revealed a decrease in miR397 levels after excess B-treatment. Excess B treatment increased the transcription of laccase7 (LAC7), the target of miR397. This treatment also increased laccase activity significantly establishing that LAC7 plays a vital role in protein biosynthesis [102]. Furthermore, HTIS identified miRNAs and their differential expression patterns in leaves of *C. sinensis* (tolerant) and *C. grandis* (intolerant) treated with B. Molecular and anatomical approaches were used to verify candidate miRNAs [103]. After B-toxic treatment, 51 miRNAs were differently expressed in *C. grandis*, whereas 20 miRNAs were significantly expressed in *C. sinensis*. In B-treated *C. sinensis* leaves, miR395a was significantly upregulated, but miR397a was downregulated. The 5′-RACE study of four *ARF* genes and two laccase (*LAC*) genes revealed that they are miR160 and miR397’s true targets. A downregulation of LAC17, whereas an upregulation of LAC4, caused poor vessel development in *C. grandis*. In *C. sinensis* vessels, secondary deposition of cell-wall polysaccharides occurred. miR397a plays a vital role in woody cell wall formation by targeting LAC17 and LAC4, which are involved for secondary cell wall synthesis [103]. Similarly, the HTIS of seeds of ‘Xuegan’ [*C. sinensis* Osbeck] revealed the upregulation of 91 and downregulation of 81 miRNAs in B-deficient leaves. There are several possible mechanisms by which miRNAs adapt to B-deficiency: (a) As a result of altered TIR1 expression, and alterations in the expression of miR393, miR160, and miR3946, deficient auxin signaling results in diminished plant growth and development; (b) upregulation of NACs and the maintenance of leaf phenotype through miR159, miR782, miR3946, and miR7539; (c) downregulation of the expression of miR164, miR6260, miR5929, miR6214, miR3946, and miR3446 induced stress responses and antioxidant system; (d) reducing the expression of miR5037-targeted major facilitator superfamily protein genes, thus limiting B export from plants. A plant’s tolerance to B-deficiency might also be influenced by the downregulation of miR408, which regulates Cu homeostasis and enhances SOD activity [104]. In another Citrus study utilizing HTIS, from root tissues of tolerant *C. grandis* and intolerant *C. grandis* treated with B toxicity, distinct miRNA expressions were discovered [105]. Overall, in response to B poisoning, 37 miRNAs were differently expressed. 5′-RACE and qRT-PCR results showed that *MYB* gene, a *SCARECROW-like protein* gene, and a cation transporting *ATPase* gene were the targets of miR319, miR171, and miR396g-5p, respectively. When SCARECROW expression is maintained in B-treated Citrus roots, stem cells and the endodermis remain specified, allowing root extension under B-toxic circumstances. Downregulation of MYB owing to miR319 upregulation in *C. grandis* roots treated with B-toxicity might substantially alter root system architecture by diminishing root tips. miR319 and miR171 appear to play a critical role in Citrus tolerance to long-term B toxicity by targeting MYB and SCARECROW, both of which are involved in root growth and development [105].

B-toxicity was shown to have a substantial effect on genes encoding jasmonate (JA), ethylene, and a cell wall modifier in wheat. Arabidopsis was investigated under hazardous B circumstances to evaluate the expression levels of miRNAs (miR172 and miR319) that target JA and ethylene-related TFs, and miR397, which targets laccase. The expression of mature miRNA was analyzed by stem-loop qRT-PCR [106]. Mild B toxicity (condition 1B) significantly increased the expression levels of miRNAs targeting TFs involved in JA and ethylene metabolism, but not severe B toxicity (condition 3B). Arabidopsis was most significantly regulated by miR172 and miR319 genes. miR397 expression did not significantly change under B toxicity, demonstrating that laccase-induced modifications to the cell wall are not regulated post-transcriptionally. As well as targeting TFs related to JA and ethylene metabolism through miRNAs, Arabidopsis can detect oxidative stress and adapt to B toxicity by producing these miRNAs [106]. To address disparities in performance under high levels of B, the same author group examined the transcriptional regulation of miR319, miR172, and miR398, and their likely target genes, in Bolal-2973 (B-tolerant) and Atay-85 (B-sensitive) wheat cultivars. Expression levels of miR398 in *Atay*, and *Cu/ZnSOD* gene expression, were higher than in Bolal after exposure to toxic B. As a result, both toxic B and ethylene-related miRNAs (miR172 and miR319) showed stable levels in wheat cultivars, exhibiting that it may induce leaf senescence. Atay, a sensitive cultivar, was only affected by miR172 targeting TF-TOE1. On the other hand, miR319 targeted MYB3 in both cultivars, and MYB3 expression was significantly boosted upon B toxicity. Additionally, the authors determined the Arabidopsis orthologs of the wheat miRNA targets. GO enrichment analyses of miRNA targets were conducted to identify functional protein association networks. According to new research, *Triticum aestivum* is home to several genes whose targets are miR172, miR319, and miR398. Furthermore, due to interaction amongst miRNA-mediated post transcriptional pathways, miR172, miR319, and miR398 are extremely susceptible to nutritional shortages or toxicities, such as those of Fe, P, B, S, and Cu [107].

### 2.7. miRNAs and Magnesium Stress

The chlorophyll molecule contains the elements magnesium (Mg) and chloride [108]. More than 300 enzymes, including ribulose-1,5-bisphosphate carboxylase, ATPase, protein kinases, phosphatases, glutathione synthase, and many others, use Mg as a cofactor; and it is an allosteric modulator for a variety of physiological and biochemical processes, including photosynthesis, respiration, organic acid metabolism, and carbohydrate partitioning between source and sink organs [109,110]. There is a widespread Mg deficiency in citrus crops, which affects their productivity and quality [111]. Despite this, there are limited data on miRNAs in higher plants that respond to Mg shortage (Table 1). Mg-deficient *C. sinensis* leaves revealed 75 upregulated and 71 downregulated miRNAs [112]. In addition to their amazing metabolic flexibility, leaf miRNAs’ adaptive responses to Mg shortage are believed to entail numerous aspects: (a) increasing stress-related genes by inhibiting miR3946 and miR5158 expression while increasing miR395, miR1077, miR1160, and miR8019 expression; (b) improving cell transport by inhibiting miR3946 and miR5158 expression while increasing miR395, miR1077, miR1160, and miR8019 expression; (c) repressing miR158, miR5256, and miR3946 to induce lipid metabolism. The researchers also discovered a number of candidate miRNAs that may have roles in Mg deficiency tolerance (i.e., miR7812, miR8019, miR6218, miR1533, miR6426, miR5256, miR5742, miR5561, miR5158, and miR5818). These findings add to our understanding of how plants respond to Mg shortage [112]. Mg-starved roots also exhibited increased expression of 101 miRNAs and decreased expression of 69 miRNAs via HTIS. Several factors contributed to citrus roots’ adaptation to Mg deficiency, including: (a) inhibiting root respiration (via miR158 and miR2919) by downregulating related miRNAs (miR780); (b) reducing inflammatory mediators by decreasing miRNAs (miR780, miR6190, miR1044, miR5261, and miR1151); (c) increasing the expression of transport-related genes by regulating miR6190, miR6485, miR1044, miR5029, and miR3437 expression; (d) controlling miR544, miR5261, miR1151, and miR5029 expression to increase protein ubiquitination; (e) regulating miR5261, miR6485, and miR158 expression to contribute to root development; and (f) regulating transcriptional regulation of DNA repair by regulating miR5176 and miR6485 [113].

Plant growth and development can be increased by using nanoparticles (NP) at the right time. Plants utilize a variety of mechanisms, including gene expression and microRNAs, to control stress responses and maintain homeostasis. In a recent study, with varied amounts of treatment, MgO-NP altered gene expression, miRNA levels, cell morphology, chlorophyll content, and physiological changes in *Ananas comosus var bracteatus*. Four grams of MgO-NP significantly increased miR396 and miR398 expression, while simultaneously repressing *RHS12* and *XTH* expression [114].

### 2.8. miRNAs in Manganese Stress

Plant growth is dependent upon the enzymatic processes mediated by manganese (Mn), which is an inorganic catalyst. Mn is an essential component of plants’ biochemical processes, such as photosynthesis, respiration, and nitrogen assimilation. Moreover, it is required for pollen germination, the growth of pollen tubes, root cells’ elongation, and protection of roots from root pathogens [115]. Only a few studies have been conducted on Mn toxicity in plants. Further research is required to determine whether miRNAs and their targets can modulate Mn toxicity. Therefore, it is difficult to find comprehensive information about the posttranscriptional regulation of Mn toxicity. Researchers used both miRNA microarray hybridization and qRT-PCR to identify miRNAs responsive to Mn in the common bean (*P. vulgaris*). According to the study, 37 miRNAs showed differential expression under abiotic and Mn stress conditions. Mn poisoning caused the activation of 11 miRNAs and the inhibition of 11 others. miR1508, miR1515, miR1510/2110, and miR1532 were revealed to be new Mn-responsive miRNAs. Leucine-rich repeat-resistant proteins, receptor kinase proteins, and calcium-dependent protein kinases were identified as key targets among Mn-responsive miRNAs [116]. Additionally, a recent study showed Arabidopsis miRNAs that respond in cell growth, nutrient homeostasis, and ion transport, differentially expressed under Mn stress conditions [117].

### 2.9. miRNAs and Iron Stress

An essential ingredient in hemoglobin is iron (Fe) [118]. Fe is usually inaccessible to plants due to its inadequate solubility in neutral and alkaline soils. Fe toxicity in plants, on the other hand, may be prompted in acidic soils by anaerobic circumstances [119]. In Fe-deficient conditions, plants control the transcriptional and post-transcriptional levels of the molecular cis-elements. In plants, Fe is involved in chlorophyll synthesis and maintenance of chloroplast structure and function [120]. Plants’ responses to Fe deficiency have recently been proposed to be mediated by miRNAs [121]. Eight unique miRNAs (miR159, miR169, miR172, miR173, and miR394) from five families were preserved in an Fe shortage in Arabidopsis, and indicated being beneficial and differently produced in response to Fe deprivation [122] (Table 1). The promoters of several *iron-deficiency-inducible (IDE)* genes contain *IDE1* and *IDE2*, which respond to Fe deficiency. Surprisingly, numerous Arabidopsis miRNAs with *IDE1/IDE2* patterns in their supporters have been found to be sensitive to Fe deficiency [123]. In Arabidopsis treated with Fe deficiency, seven miRNAs from eight families displayed markedly different expression levels (miR172, miR158, miR163, miR165, miR166, miR397, and miR398) [123]. Similarly, it was observed that miR408 was overexpressed in Fe-deficient Arabidopsis plants [124]. Furthermore, using northern blot analysis and microarrays, in the common bean, multiple miRNAs were found to respond to Fe deficiency, including miR167, miR397, miR398, and miR408 [116].

### 2.10. miRNA and Other Nutrients

Plant growth and development are increasingly reliant on zinc (Zn) as a micronutrient [125]. A large percentage of the world’s soil is deficient in Zn [126]. Many metabolic reactions in plants are driven by Zn, which is an important component of many enzymes. Plants would cease to grow and their development would be hindered without certain enzymes. Zn-deficient plants produce significantly less carbohydrates, proteins, and chlorophyll [127]. Only a few studies have addressed miRNAs’ role in plants responding to Zn deficiency (Table 1). For example, a study using Solexa sequencing identified several miRNAs that responded to Zn deficiency in *Brassica juncea* roots [128]. Both Zn-deficient and control roots of *B. juncea* contained 101 members of 21 conserved miRNA families. Plants with Zn deficiency and control plants showed differential expression of 15 miRNAs from nine miRNA families. The Zn-deficient roots of *B. juncea* showed upregulated expression of 13 miRNAs, while miR399b and miR845a appeared to be downregulated. Abiotic stress causes *B. juncea* roots to modulate these miRNAs, which control the phytohormone response, plant development, and abiotic stress responses. As a consequence of these discoveries, we now have a better understanding of how miRNA regulates the Zn-deficiency response in plants, and how this affects plant growth and development [128].

Peanuts are the most widely grown cash crop among leguminous plants, owing to their high protein content and ability to produce oil. They are extensively grown in tropical and subtropical climates [129]. Quality and yield are directly impacted by the development of peanut embryos. Geocarpic plants, such as peanuts, go through a complicated embryo development process that involves a number of gene regulation mechanisms at both the transcriptional and posttranscriptional levels, and these are easily influenced by soil components, such as calcium (Ca) [130]. Ca in the soil (pegging zone) is essential for the development of embryos. The yield and quality of peanuts are severely reduced when calcium is deficient [131]. Deficient Ca also adversely affects seed viability and germination after a season. Peanut embryos are born dead as a result of severe soil calcium deficiency. A recent study focused on analyzing the sRNAs in early peanut embryos with the aid of a recently established platform for the sequences of genomes of wild peanut species (Table 1). Twelve peanut-specific miRNA families were found to host 29 known miRNAs and 132 potential novel miRNAs. Of the identified miRNAs, 87 showed differential expression during early embryo development in the presence of Ca deficiency or sufficiency, and 117 target genes also showed differential expression [132]. Twenty miRNAs differentially expressed 52 target genes according to an integrated analysis of miRNAs and transcriptome expression. A comparison of gene chip analysis and transcriptome sequencing revealed some targets that were differentially expressed. These results indicate that miRNAs actively modulate the expression of genes associated with embryo development, such as *TCP3 (Teosinte branched1/Cycloidea/Proliferating cell factor), AP2 (Apetala 2)*, *EMB2750 (embryo-defective)*, *GRFs, cytochrome P450* (*CYP707A1* and *CYP707A3*), which conveys ABA, and BR1, which transports brassinosteroids (BRs). Both miRNAs and their related target genes are thought to participate in peanut embryo abortion in response to Ca deficiency. These findings establish miRNA-mediated regulatory mechanisms implicated in embryo abortion in the absence of Ca in peanut embryos [132].

**Table 1:** Nutrient responsive miRNAs: their regulations and target functions in plants.

miRNAs	Targets	Plant Species	Target Function	Regulation	Nutrients											References
					N	P	K	S	Cu	Fe	B	Mg	Mn	Zn	Ca	
miR156	SPLs	*Camellia sinensis*	Shoot development	Up	√											[133]
	SPLs	*Lupinus angustifolius*	Seed development	Up		√										[134]
	SPLs	*Brassica napus*	Seed maturation	Up				√								[135]
	NAC4, ARF2, AFB3	*Arachis hypogaea*	Root development	Up	√											[76]
miR157	SPLs	*Citrus sinensis*	Root development	Down							√					[101]
miR158	BZIP	*Solanum lycopersicum*	Plant development	Up		√										[136]
	AP2, SBP, NAC, MYB	*Arabidopsis thaliana*	Plant development	Up						√						[123]
	AP2, SBP, NAC, MYB	*Vitis vinifera*	Plant growth and development	Up					√							[96]
	SPLs	*Citrus sinensis*	Respiration management	Up								√				[113]
	FUT1	*Brassica juncea*	Plant development and abiotic stress response	Up										√		[128]
miR159	MYBs and TCPs	*Cucumis sativus*	Plant development	Up	√											[137]
	MYBs	*Betula luminifera*	Root development	Down		√										[138]
	NRAMP4	*Oryza sativa*	Root development	Up						√						[139]
	MYBs	*Triticum aestivum*	Root development	Up	√											[140]
miR160	ARFs	*Arabidopsis thaliana*	Root development, signal transduction	Up	√											[29]
	SPLs	*Arachis hypogaea*	Root development	Down	√											[76]
	GRFs	*Triticum aestivum*	Signal transduction	Down			√									[74]
	ARFs	*Brassica juncea*	Hormone signaling	Up										√		[128]
miR162	DCLs	*Zea mays*	Flower development	Up	√											[28]
	DCLs	*Oryza sativa*	Iron homeostasis	Up						√						[141]
miR164	NAC	*Arabidopsis thaliana*	Leaf senescence	Up	√											[51]
	SPLs	*Arachis hypogaea*	Root development	Down	√											[76]
	TCA cycle	*Arachis hypogaea*	Potassium stress	Up/Down			√									[76]
miR165	HD-ZIP	*Hordeum vulgare*	Root development	Up							√					[99]
miR166	HD-ZIP	*Populus tomentosa*	Shoot development	Down	√											[142]
miR168	AGOs	*Cucumis sativus*	Signal transduction	Down	√											[137]
	AGOs	*Solanum lycopersicum*	Root development	Down			√									[77]
	AGOs	*Oryza sativa*	Root development	Up/Down						√						[141]
miR169	HAP2	*Arabidopsis thaliana*	Nitrogen homeostasis, stress response	Down	√											[37]
	HAP2	*Sorghum bicolor*	stress response	Down			√									[143]
	Pentose pathway	*Triticum aestivum*	Potassium stress	Up/Down			√									[74]
	CAAT TFs	*Phaseolus vulgaris*	Leaf formation	Up									√			[116]
	CAAT TFs	*Brassica juncea*	Plant development	Up										√		[128]
miR171	SCLs	*Arabidopsis thaliana*	Root development	Down	√											[29]
	SCLs	*Oryza sativa*	Root development	Up/Down						√						[141]
	Signaling pathways	*Taxus chinensis*	Root development	Up/Down				√								[144]
	SCARECROW-like protein	*Citrus sinensis*	Root development	Up							√					[105]
miR176	MLH1	*Citrus sinensis*	Respiration management	Up								√				[113]
miR319	TCPs	*Cucumis sativus*	Shoot development	Down	√											[137]
	TCPs	*Hordeum vulgare*	Potassium homeostasis	Down			√									[72,73]
	Signaling pathways	*Taxus chinensis*	Root development	Up/Down				√								[144]
	MYBs	*Citrus sinensis*	Root development	Up							√					[105]
	ethylene-related TFs	*Arabidopsis thaliana*	oxidative stress-adaptive responses	Up							√					[106]
	MYBs	*Triticum aestivum*	Nutrient stress response	Up							√					[107]
	TCPs	*Brassica juncea*	Plant development	Up										√		[128]
miR390	NAC4, ARF2, and AFB3	*Arachis hypogaea*	Lateral root development	Up	√											[76]
	Serine/threonine protein kinase	*Phaseolus vulgaris*	Nodule formation	Up									√			[116]
miR393	Auxin receptors	*Zea mays*	Development of roots	Up	√											[42]
	SPLs	*Arachis hypogaea*	Root development	Down	√											[76]
	Auxin signaling	*Oryza sativa*	Development of Auxiliary buds	Up/Down	√											[145]
	Basic helix-loop-helix (bHLH)	*Brassica juncea*	Plant development	Up										√		[128]
miR394	F-box	*Oryza sativa*	Shoot development	Up	√											[146]
	F-Box	*Brassica juncea*	Plant development	Up										√		[128]
miR395	ATP sulfurylase; Sulfate transporters	*Cucumis sativus*	Sulfur metabolism	Down	√											[137]
	Ca^2+^ signaling pathway	*Sorghum bicolor*	Potassium stress	Up/Down			√									[143]
	ATP sulfurylase; Sulfate transporters	*Arabidopsis thaliana*	Nutrient stress response	Down									√			[117]
miR396	GRF	*Oryza sativa*	Leaf development	Down	√											[147]
	GRF	*Hordeum vulgare*	Potassium homeostasis	Down			√									[72,73]
	GRF	*Oryza sativa*	Seedling growth	Down	√											[147]
	GRF	*Phaseolus vulgaris*	Plant development	Up							√					[100]
	RHS12	*Ananas comosus var. bracteatus*	Plant development	UP								√				[114]
	GRF	*Phaseolus vulgaris*	Nodule formation	Up									√			[116]
miR397	Laccases	*Zea mays*	Lignin synthesis/Copper homeostasis	Down	√											[42]
	Laccases	*Arabidopsis thaliana*	Metabolic processes	Down				√								[40]
	Laccases	*Poncirus trifoliata*	Stress response	Up							√					[102]
	Laccases	*Citrus sinensis*	Cell wall biosynthesis	Down							√					[103]
miR398	CSD; COX5b-1; CCS1	*Medicago sativa*	Oxidative stress/Copper homeostasis	Down	√											[148]
	SOD	*Arabidopsis thaliana*	Metabolic processes	Down				√								[40]
	SPLs	*Arabidopsis thaliana*	Metabolic processes	Down					√							[93]
	GATA type zinc finger TFs	*Phaseolus vulgaris*	Regulate light-sensitivity	Down							√					[100]
	XTH	*Ananas comosus var. bracteatus*	Plant development	UP								√				[114]
	Cu/Zn SOD	*Brassica juncea*	Plant development	Up										√		[128]
	Unknown	*Arachis hypogaea*	Embryo development	Up											√	[132]
miR399	UBC24/PHO2	*Zea mays*	Phosphate homeostasis	Down	√											[149]
	PHO2	*Sorghum bicolor*	Plant development	Down			√									[143]
	PHO2	*Arabidopsis thaliana*	Metabolic processes	Down				√								[40]
	Ubiquitin conjugase E2	*Arabidopsis thaliana*	Nutrient stress response	Down									√			[117]
	PHO2	*Brassica juncea*	Phytohormone response	Down										√		[128]
miR401	Unknown	*Citrus sinensis*	Leaf development	Down							√					[104]
miR408	Laccases; plantacyanin	*Zea mays*	Lignin synthesis/Copper homeostasis	Down	√											[42]
	SOD	*Arabidopsis thaliana*	Metabolic processes	Down				√								[40]
	SPLs	*Arabidopsis thaliana*	Metabolic processes	Down					√							[93]
	laccases	*Arabidopsis thaliana*	Iron homeostasis	Up/Down						√						[124]
	Basic blue copper protein	*Phaseolus vulgaris*	Leaf development	Up									√			[116]
miR482	Ca^2+^ signaling pathway	*Sorghum bicolor*	Potassium stress	Up/Down			√									[143]
miR485	Unknown	*Brassica juncea*	Phytohormone response	Down										√		[128]
miR528	Pytocyanin; CSD	*Agrostis stolonifera*	Oxidative stress	Down	√											[150]
miR535	CSD	*Vitis vinifera*	Copper homeostasis	Down					√							[96]
miR780	Na+/H+ antiporter	*Arabidopsis thaliana*	Export of Sodium ion	Up	√											[29]
miR781	MCM2 (At1G44900)	*Arabidopsis thaliana*	Nutrient stress response	Up									√			[117]
miR826	AHP2	*Populus* spp.	Glucosinolate synthesis	Up	√											[151]
	Signaling pathways	*Taxus chinensis*	Root development	Up/Down				√								[144]
	Alkenyl hydroxalkyl producing 2	*Arabidopsis thaliana*	Nutrient stress response	Up									√			[117]
miR827	Ubiquitin E3 ligase NLA	*Zea mays*	phosphorus metabolism/Nitrogen	Down	√											[42]
miR842	Jacalin lectin family	*Zea mays*	Unknown	Up	√											[42]
miR843	Kinesin motor-related	*Citrus sinensis*	Root development	Up							√					[101]
miR846	Jacalin lectin family	*Zea mays*	Unknown	Up	√											[42]
miR857	Laccases	*Zea mays*	Lignin synthesis/Copper homeostasis	Down	√											[42]
	Signaling pathways	*Taxus chinensis*	Root development	Up/Down				√								[144]
miR1432	Unknown	*Sorghum bicolor*	Root development	Up/Down	√											[143]
miR2004	PHD finger family proteins	*Hordeum vulgare*	Root development	Up							√					[99]
miR3511	ROS	*Arachis hypogaea*	Embryo development	Down											√	[132]
miR3515	ROS	*Arachis hypogaea*	Embryo development	Down											√	[132]
miR4351	Unknown	*Citrus sinensis*	Leaf devlopment	Up								√				[112]
miR5026	Unknown	*Arabidopsis thaliana*	Nutrient stress response	Up									√			[117]
miR5029	PBA1	*Citrus sinensis*	Respiration management	Up								√				[113]
miR5051	Unknown	*Hordeum vulgare*	Root development	Up							√					[99]
miR5261	Mrel1	*Citrus sinensis*	Respiration management	Down								√				[113]
miR5266	Unknown	*Citrus sinensis*	Leaf development	Up							√					[104]
miR5564	Unknown	*Sorghum bicolor*	Shoot development	Up/Down		√										[143]
miR5565	Unknown	*Sorghum bicolor*	Stress response	Up/Down			√									[143]
miR5595	MES	*Arabidopsis thaliana*	Nutrient stress response	Up									√			[117]
miR5832	Unkonwn	*Citrus sinensis*	Leaf development	Up								√				[112]
miR6485	VALRs	*Citrus sinensis*	Respiration management	Up								√				[113]

Abbreviations: SPLs (SQUAMOSA promoter-binding protein-like), AHP2 (alkenyl hydroxalkyl producing 2), CSD (cold-shock domain), PHO2 (phosphate starvation 2), SOD (superoxide dismutase), COX (cyclooxygenases), GRF (growth response factors), ARF (Auxin response factors), DCL (dicer-like), MYB (myeloblastosis), TCA (tricarboxylic acid cycle), NRAMP4 (natural resistance-associated macrophage protein 4).

## 3. Conclusions and Prospects

Since the discovery of miRNA, many nutrient-receptive miRNAs have been uncovered using diverse methodologies. However, mechanical information about the functions of nutrient-receptive miRNAs still requires further understanding and research. Similarly, more investigations related to these specified miRNAs and their genetic targets will provide us with better insights into the monitoring networks of plants for nutrient stress. Although recent advancements in high-throughput sequencing have enabled us to quickly classify the miRNAs, it is unclear whether these miRNAs, such as miR172 and miR398, regulate targets through translational inhibition or employ some other mechanisms. Moreover, focus should be shifted towards understanding the involvement of miRNAs in nutritional stress responses via paying special attention to phenotypical and physiological changes caused by these regulations. Nutritional stress can also adversely affect transposon-derived RNAs and tRNA-derived RNAs, in addition to miRNAs; however, their physiological roles are unknown. To ensure future food security, miRNA-based strategies could be vital, in order to create crop varieties with higher productivity and resistance to abiotic and biotic stress.

## Data Availability

Not applicable.
